# Melatonin improves the quality of maternally aged oocytes by maintaining intercellular communication and antioxidant metabolite supply

**DOI:** 10.1016/j.redox.2021.102215

**Published:** 2021-12-17

**Authors:** Hui Zhang, Chan Li, Dongxu Wen, Ruoyu Li, Sihai Lu, Rui Xu, Yaju Tang, Yidan Sun, Xiaoe Zhao, Menghao Pan, Baohua Ma

**Affiliations:** a College of Veterinary Medicine, Northwest A&F University, Yangling, Shaanxi, People's Republic of China; b Key Laboratory of Animal Biotechnology, Ministry of Agriculture, Yangling, Shaanxi, People's Republic of China

**Keywords:** Melatonin, Reproductive aging, Transzonal projections, Cumulus cells-oocyte communication, Reactive oxygen species, Endoplasmic reticulum stress

## Abstract

In mammalian ovaries, oocytes are physically coupled to somatic granulosa cells, and this coupling is crucial for the growth and development of competent oocytes as it mediates the transfer of metabolic support molecules. However, aging-mediated dysregulation in communication between the oocytes and granulosa cells affects the oocyte quality. In the present study, we examined the defected germline-soma communication and reduced mRNA levels encoding key structural components of transzonal projections (TZPs) in maternally aged oocytes. Oral administration of melatonin to aged mice substantially increased TZPs and maintained the cumulus cells-oocyte communication, which played a central role in the production of adequate oocyte ATP levels and reducing the accumulation of reactive oxygen species (ROS), apoptosis, DNA damage, endoplasmic reticulum (ER) stress and spindle/chromosomal defects. This beneficial effect of melatonin was inhibited by carbenoxolone (CBX), a gap junctional uncoupler, which disrupts bidirectional communications between oocyte and somatic cells. Simultaneously, melatonin significantly increased the mRNA and protein levels corresponding to genes associated with TZPs and prevented TZP retraction in in vitro-cultured cumulus-oocyte complex (COCs). Furthermore, we infused melatonin and CBX into the COCs in vitro culture system and monitored the levels of nicotinamide adenine dinucleotide phosphate (NADPH) and glutathione (GSH) in cumulus cells and oocytes. Notably, COCs treated with melatonin demonstrated improved NADPH and GSH levels. Of note, CBX was capable of reducing NADPH and GSH levels, aggravated the ROS accumulation and ER stress. Collectively, our data demonstrate the role of melatonin in preventing age-associated germline-soma communication defects, aiding the relay of antioxidant metabolic molecules for the maintenance of oocyte quality from cumulus cells, which have important potential for improving deficient phenotypes of maternally aged oocytes and the treatment of woman infertility.

## Highlights

• Communication between the oocytes and cumulus cells declines with age.
• Melatonin repair of aging-induced dysregulation in communication between the oocytes and cumulus cells.
• Melatonin preventing age-associated metabolic co-dependence defects of oocytes and cumulus cells.
• Melatonin attenuates mitochondrial dysfunction and endoplasmic reticulum stress.

## Graphical abstract

**Figure:**
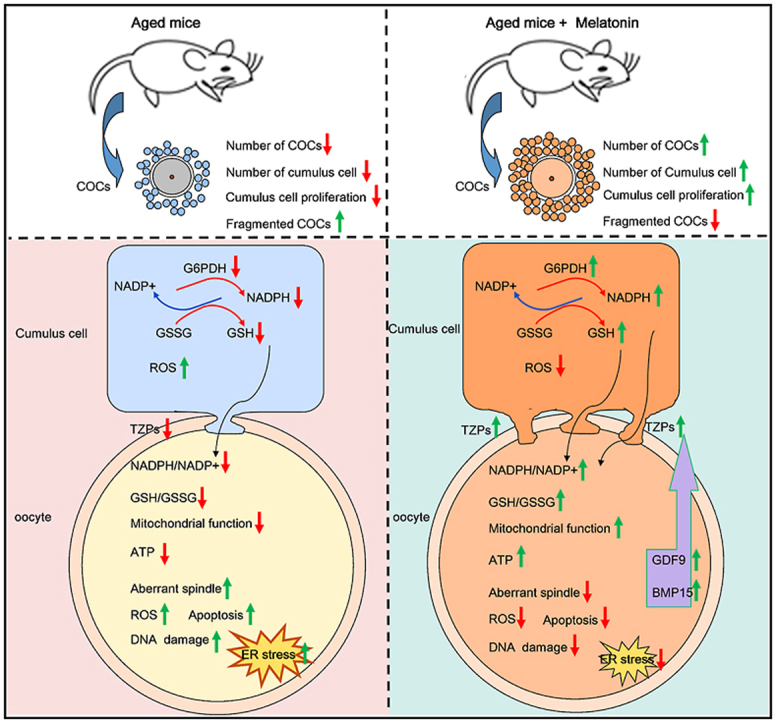

## 1 Introduction

Declining oocyte quality associated with reproductive aging is one of the key rate-limiting factors of the fertility of female mammals. The oocyte quality of women begins to diminish in the early 30s and declines dramatically after the age of 35 [1,2]. The aging-related damage in the human female reproductive system is more critical than that observed in other organ systems [3]. The ovarian functional decline and oocyte quality decay caused by the oxidative damage during ovarian aging significantly affect female fertility [4,5].

A novel defect associated with reproductive aging, which impairs the interaction between the oocyte and its follicular environment, has been identified recently [6]. However, the associations among aging, impaired germline-soma communication and infertility remain unclear. During the mammalian reproductive cycle, as the oocyte grows and matures, oocyte developmental competence is significantly dependent on the communication with adjacent somatic cells [[7], [8], [9], [10]]. Germ-soma contact and communication are mediated by transzonal projections (TZPs) that extend from the cumulus granulosa cells. These projections are specialized actin-rich filopodia that provide a means of conductance of small regulatory factors and large molecules, including RNA and metabolites, to the oocyte, which play critical roles in promoting the oocyte growth and quality [[11], [12], [13]]. The majority of glucose consumed by cumulus-oocyte complex (COCs) during maturation is metabolized in the cumulus cells, with an insignificant or no amount consumed by the oocyte [14,15]. Germ-soma communications have been shown to play a central role in maintaining adequate oocyte ATP levels by relaying the metabolic support from cumulus cells [16]. Although detailed molecular mechanisms underlying ovarian aging are poorly understood, defective germline-soma communication may cut off the influx of the metabolites and information-relaying molecules essential for the promotion of oocyte cytoplasmic maturation [6]. Ovarian aging leads to the gradual accumulation of reactive oxygen species (ROS), which are thought to be the major drivers underpinning DNA damage and mitochondrial de-regulation [17]. Impaired mitochondrial function further promotes the production of ROS [5]. Free radical scavengers and antioxidants are widely perceived as the contributing factors that delay ovarian aging. Nicotinamide adenine dinucleotide phosphate (NADPH) plays an important role in the regeneration of the antioxidant glutathione (GSH) that neutralizes ROS [16,18]. One of the most intriguing aspects of COC metabolism is that NADPH generated via the pentose phosphate pathway (PPP) in cumulus cells is transferred to the oocyte via gap junctions [16]. A defective germ-soma communication may lead to metabolic disorders and mitochondrial dysfunction by compromising the transfer of antioxidants.

Melatonin (5-methoxy-N-acetyltryptamine) is an endogenous hormone that is primarily released by the pineal gland to modulate many important physiological reactions [19]. Melatonin modulates mitochondria-related functions and strengthens its antioxidant defense systems by scavenging most toxic free radicals [20]. This scavenger property enables them to protect cellular membranes, mitochondria, and the electron transport chain from oxidative injury [21]. Melatonin can be synthesized in the follicular granulosa cells and oocytes. The melatonin biosynthesis is mediated by the mitochondria to resist free radicals [[22], [23], [24]]. Unfortunately, mitochondrial dysfunction caused by the aging and several diseases will jeopardize melatonin production and cause more serious oxidative damage [25]. Maternal aging leads to the reduction of melatonin both in blood serum and follicular fluid. The maintenance of high melatonin concentration in the follicular fluid microenvironment exerts positive effects on the oocyte quality. Notably, insufficient amounts of melatonin in follicular fluid are highly correlated with advanced maternal age-related meiotic defects [26,27]. Maintenance of high concentrations of melatonin hampers oocyte maturation in pre-ovulatory follicles by resisting the oxidative stress that accompanies free oxygen radicals generated during ovulation [28]. In addition, this potent antioxidant released by the pineal gland is highly effective at preventing age-related oxidative stress and reproductive system disorders [29,30].

Melatonin has been shown to induce microfilament organization, filopodia formation and elongation [31]. However, to date, it remains to be determined whether melatonin rescues the decline of oocyte quality during the aging process by influencing the contact and communication between granulosa cells and oocytes. In the present study, we hypothesized that melatonin might be involved in mediating the oocyte-granulosa cell cross-talk to maintain the metabolic co-dependence of the two components in aged mice. Our results show that deficient granulosa cell-oocyte communication was caused by the reduced number of TZP connections. Melatonin administration in aged mice remarkably increased the number and density of TZPs in COCs and prevented TZP retraction in vitro, which maintained granulosa cell-oocyte communication, thereby allowing an increase in the trafficking of the metabolites and information-relaying molecules essential to oocyte maturation and quality.

## 2 Materials and methods

### 2.1 Animals and feeding treatment

The experimental protocols and mice handling procedures were reviewed and approved by the Institutional Animal Care and Use Committee of the College of Veterinary Medicine, Northwest A&F University (No.2018011212). Young (6∼8-week-old) and ovarian aged (50∼60-week-old) Kunming female mice were obtained from the Experimental Animal Center of the Xi'an Jiaotong University. All mice used in this study were maintained under a light and temperature-controlled environment (12 h light/dark cycle, 20∼25 °C) and provided with ad libitum access to food and water. Aged mice were randomly divided into two groups. One group was administered water orally, and the other group was subjected to oral administration of melatonin (30 mg/kg body weight) at 5:00 p.m. for 28 days preceding collection and analysis of oocyte or COCs. The dose of melatonin was selected based on the published reports [32,33]. For some experiments, carbenoxolone disodium (CBX, TargetMol, Boston, MA), a gap junction blocker, was dissolved in 0.9% saline and administered (10 mg/kg body weight) intraperitoneally (i.p.). Controls were injected i.p. with equal volumes of 0.9% saline. CBX or saline (i.p.) injections were administered 48 h prior to the oocyte or COCs collection.

### 2.2 Collection and culture of oocytes and COCs

Female mice were superovulated using an intraperitoneal injection of 5IU equine chorionic gonadotropin (Serum Gonadotrophin for Injection, Ningbo Second Hormone Factory, Zhejiang, China), and ovaries were collected after 44–46 h. The ovaries were placed in a handling minimum essential medium (MEM) α supplemented with 3 mg/ml of bovine serum albumin (BSA). The well-developed graafian follicles were punctured with 30-gauge needles, and GV oocytes or COCs were collected. OOX cumulus cells were obtained by microsurgically removing oocyte from the COCs as described previously. A microinjection apparatus (Narishige ON399D, Japan) was used to guide the holding and injecting micropipettes [34].

GV oocytes or COCs were cultured in MEM-α (Life Technologies, New York, USA) supplemented with 3 mg/ml of BSA and 0.23 mM pyruvate at 37 °C in an atmosphere containing 5% CO_2_ with maximum humidity. For in vitro supplementation, fully grown oocytes or COCs were cultured in a maturation medium containing various concentrations of melatonin. Melatonin was dissolved in absolute ethanol and diluted with a maturation medium to obtain the final concentration. In some experiments, the culture medium was supplemented with 1 μM CBX, 10 μM SB431542 (Sigma), and 100 nM LDN193189 (Sigma). The reagent solution was freshly prepared for each experiment with a final ethanol concentration of 0.01% or DMSO concentration of 0.01%. Controls were always treated with the same amount of ethanol or DMSO, unless stated otherwise.

### 2.3 Evaluation of cumulus cells per oocyte

Cumulus cells were stripped from the COCs by repeated mechanical pipetting with a 200 μL pipette in 1 mL MEM-α medium. The cumulus cells were counted using a hemocytometer. To obtain the number of cumulus cells per COC, the number of cumulus cells was divided by the number of COCs.

### 2.4 BrdU incorporation assay

Cumulus cell proliferation was assessed by BrdU incorporation. Mice were injected with 100 mg BrdU/kg body weight 2 h prior to ovary collection. COCs were collected and fixed for 1 h in 4% paraformaldehyde. After 3 washes with PBS containing 0.1% Tween-20, COCs were permeabilized with 0.5% Triton X-100 for 20 min and blocked with 3% BSA in PBS for 30 min, then the COCs were incubated overnight with Rabbit anti-BrdU (100-fold dilution; Sigma) primary antibody. After thorough washing, the COCs were incubated for 1 h with Alexa Fluor 594-conjugated donkey anti-Rabbit secondary antibody (200-fold dilution; Thermo Fisher Scientific). The percentage of BrdU positive cumulus cells in relation to DAPI positive cells was determined by a confocal scanning laser microscopy (Nikon A1R-si) and the NIS Elements Basic Research software.

### 2.5 Staining and analysis of TZPs

For TZP analysis, oocytes were washed in 0.1% Polyvinyl alcohol (PVA)-PBS (wt/vol), fixed in 4% paraformaldehyde(PFA)-PBS for 30 min at 37 °C, blocked in 1 mg/mL BSA-PVA-PBS for 1 h, and stained by fluorescein isothiocyanate labeled phalloidin (2 μg/mL) at 37 °C for 2 h in the dark. To quantify the number of actin-TZPs, a confocal optical section was obtained at the equatorial plane of the oocyte using Image-Pro-Plus 6.0 software (Media Cybernetics).

### 2.6 Immunofluorescent staining

Oocytes were fixed in 4% paraformaldehyde in PBS for 30 min at room temperature, and permeabilized with 0.5% Triton X-100 for 20 min, then blocked with 1% BSA in PBS for 1 h at room temperature. The oocytes were incubated with primary antibodies at 4 °C overnight. (rabbit polyclonal anti-γH2AX, 1:100, abcam; rabbit polyclonal anti-GRP78, 1:100, abcam), and then the oocytes were extensively washed with wash buffer (0.1% Tween 20 in PBS), probed with Alexa Fluor® 488 goat anti-rabbit antibody (1:200, Thermo Fisher Scientific, Cat #A-11008) or Alexa Fluor 594 goat anti-rabbit antibody (1:200, Thermo Fisher Scientific, Cat #A-11012) in a dark room for 1 h at room temperature. Then oocytes were counterstained with DAPI (10 μg/mL) at room temperature for 10 min. Finally, samples were mounted on glass slides and observed viewed under the confocal microscope (Nikon A1R-si).

### 2.7 Analysis of gap junctional coupling

Gap junctional coupling measurements of oocytes and cumulus cells were performed as per the protocol demonstrated in our previous report [35]. Briefly, the COCs were incubated with 1 mM calcein-acetoxymethyl (calcein-AM in Dulbecco PBS) for 15 min at 37 °C in an atmosphere containing 5% CO_2_ and humidified air to allow endogenous esterase to cleave the lipophilic acetoxymethyl. We then examined the transfer of the calcein dye from cumulus cells to the oocyte using fluorescence microscopy. The fluorescence intensity of the oocyte was dependent on the permeability of gap junction in COCs, and the integrated optical density (IOD) analysis of the oocytes and their surrounding cumulus cells was examined using Image-J software to calculate the IOD rate of cumulus/oocytes.

### 2.8 ATP measurements

Oocyte ATP content was determined using an ATP Bioluminescence Assay Kit (Beyotime Institute of Biotechnology, Shanghai, China) as per the manufacturer's protocol. Oocytes were lysed using 50 μl of ATP-releasing agent. Internal standards were prepared over the range of 0 ∼10 nM. Samples and standards were transferred to 96-well black culture plates, and emitted bioluminescence was immediately measured by reading the plates using Multimode Microplate Reader (Tecan Life Sciences). ATP levels in single oocytes were calculated based on the standard curve derived using internal standards.

### 2.9 Monitoring of ROS levels in oocytes

To determine the amount of ROS, oocytes were processed for 10 mM oxidation sensitive fluorescent probe dichlorofluorescein (DCFH) (Beyotime Institute of Biotechnology, Shanghai, China) for 30 min at 37 °C in DPBS. Then, oocytes were washed thrice with PBS containing 0.1% BSA and placed on glass slides for capturing images under a confocal microscope (Nikon A1R-si).

### 2.10 Measurement of the NADPH content and NADPH/NADP + ratio

The NADPH and NADP + contents were measured using a NADP+/NADPH assay kit (Beyotime Institute of Biotechnology) according to the manufacturer's instructions. Briefly, oocytes or cumulus cells were lysed in 100 μL of NADP+/NADPH extraction buffer on ice for 20 min. The lysate was centrifuged at 12000×*g* for 5 min at 4 °C. Then, the supernatant was transferred to the 96-well plates, and the absorbance was measured using a multimode plate reader (BioTek Epoch) at 450 nm. The amount of NADPH and total NADP+/NADPH in the sample was determined using a calibration curve. The amount of NADP+ was calculated by subtracting the amount of NADPH from total NADP+/NADPH.

### 2.11 Measurement of the GSH/GSSG ratio

The GSH and GSSG contents were measured with a GSSG/GSH Assay Kit (Beyotime Institute of Biotechnology) according to the manufacturer's instructions. Briefly, oocytes or cumulus cells were lysed in 30 μL of deproteinized buffer on ice for 10 min. The lysate was centrifuged at 12000×*g* for 5 min at 4 °C. For GSSG measurements, the samples were incubated with GSH scavenge buffer for 60 min at 25 °C to decompose GSH. Then, the samples were transferred to the 96-well plates, and the absorbance was measured using a multimode plate reader (BioTek Epoch) at 412 nm.

### 2.12 Measurement of glucose-6-phosphate dehydrogenase (G6PDH) activity

The G6PDH activities were measured with a G6PDH activity Assay Kit (Beyotime Institute of Biotechnology) according to the manufacturer's instructions. Briefly, oocytes or cumulus cells were lysed in 50 μL of G6PDH extracting buffer on ice for 30 min. The lysate was centrifuged at 12000×*g* for 10 min at 4 °C. Then, the samples were transferred to the 96-well plates with G6PDH detection buffer, and the absorbance was measured using a multimode plate reader (BioTek Epoch) at 450 nm.

### 2.13 Spindle assembly analysis

To collect MII oocytes, female mice were injected with 5 IU PMSG, followed by 5 IU of human chorionic gonadotropin (hCG) after 48 h. After 14 h, super-ovulated mice were euthanized. COCs were collected by dissecting the oviductal ampullae in DPBS. After brief incubation of COCs in 0.1% hyaluronidase, cumulus cells were removed by pipetting. MII oocytes were fixed in 4% paraformaldehyde in PBS for 30 min and permeabilized with 0.5% Triton X-100 at room temperature for 20 min. After blocking with 1% BSA in PBS for 1 h, oocytes were incubated with 1:200 Alexa Fluor® 488 Conjugate anti-α-tubulin monoclonal antibody (Cell Signaling) for 2 h at room temperature. After staining the DNA with DAPI, oocytes were mounted on glass slides and viewed under the confocal microscope (Nikon A1R-si).

### 2.14 Annexin-V staining

For Annexin-V staining, COCs or oocytes were stained using the Annexin-V Staining Kit (Beyotime Institute of Biotechnology) according to the manufacturer's instructions. The oocytes were stained with 95 μL of binding buffer containing 5 μL of Annexin-V-FITC for 20 min at room temperature. Then, oocytes were washed thrice in DPBS containing 0.1% BSA, placed on glass slides, and observed under a laser scanning confocal microscope (Nikon A1R-si).

### 2.15 Mitochondrial distribution analyses and mitochondrial membrane potential (ΔΨm) measurement

For mitochondrion distribution analyses, MII oocytes were incubated in an M2 medium containing 500 nM MitoTracker Red (Beyotime Institute of Biotechnology) for 30 min at 37 °C and an atmosphere containing 5% CO_2_. After washing thrice with M2 medium, oocytes were mounted on glass slides and observed under a laser scanning confocal microscope. Mitochondrial membrane potential (ΔΨm) measurements of oocytes were performed as per the protocol shown in our previous report [35].

### 2.16 Real-time RT-PCR analysis

Total RNA from COCs or oocytes (cumulus cells were stripped from the COCs by repeated mechanical pipetting performed using a 200 μL pipette) was extracted using MiniBEST Universal RNA Extraction Kit (TaKaRa, Dalian, China) according to the manufacturer's instructions. The total RNA was reverse transcribed to synthesize cDNA using a PrimeScript RT Master Mix reverse transcription kit (TaKaRa). Real-time RT-PCR quantitation of mRNAs was performed using TB Green™ Premix Ex Taq™ II (TaKaRa) with Applied Biosystems StepOnePlus Real-Time PCR System (Thermo Fisher Scientific, Massachusetts, USA) using the following parameters: 95 °C for 1 min, followed by 40 cycles at 95 °C for 5 s and 60 °C for 34 s. The PCR primers used in this study are shown in Table 1. Transcript levels were normalized to those of the housekeeping gene *Gapdh and Actinb*. The fold change of gene expression was analyzed using the 2^-△△Ct^ method. Only one product of the estimated size was identified by 3% agarose gel electrophoresis for each set of primers to confirm the specificity of the PCR products. Minimum information for publication of quantitative real-time PCR experiments (MIQE) RT-PCR guidelines was followed. Each experiment was repeated independently at least thrice.

**Table 1:** PCR primers.

Genes	Forward primer (5′-3′)	Reverse primer (5′-3′)	size
*Daam1*	GCGGCTGCTCAGAGTATAGAAA	AAACATGGCTTCCCTGTGTTTG	274
*Fscn1*	AGAACGCCAGCTGCTACTTT	CGAGGAATCACTACCCACCG	332
*Myo10*	TCCAGACAGACTATGGGCAGG	GGAAGCCATGTCGTCCACG	110
*Mtnr1a*	CCATTTCATCGTGCCTATG	GTAACTAGCCACGAACAGC	262
*Mtnr1b*	ATGGGCTCCTGAACCAGAAC	AGGCTAGAGAGCACCTTCCT	176
*Actinb*	GGCTGTATTCCCCTCCATCG	CCAGTTGGTAACAATGCCATGT	154
*Gapdh*	AGAGCTGAACGGGAAGCTCACT	TGCCTGCTTCACCACCTTCTTGAT	130

### 2.17 Western blot analysis

COCs were lysed in RIPA buffer (solarbio, Beijing, China) supplemented with 1 mM phenylmethylsulfonyl fluoride (solarbio, Beijing, China), a protease inhibitor, incubation on ice for 30 min. Samples were boiled at 100 °C in a metal bath for 10 min in a protein loading buffer (CoWin Biosciences, Beijing, China), and equal amounts of protein derived from 50 COCs were separated using 10% SDS-PAGE gel and transferred to polyvinylidene fluoride membranes (Millipore, Bedford, USA). After transfer, the membranes were blocked in TBST containing 3% BSA for 1 h at room temperature, followed by incubation with primary antibodies at 4 °C overnight (Rabbit anti-GAPDH, Cell Signaling, 1:2000; Rabbit anti-Fascin, Abcam, 1:2000; Rabbit anti-Myosin, Abcam, 1:1000; Mouse anti-Daam1, Santa Cruz, 1:1000, Rabbit polyclonal anti-GRP78, Abcam, 1:1000). The membrane was immersed in secondary antibodies for 1 h at room temperature, and then the membrane signals were visualized using a chemiluminescent HRP substrate reagent (Bio-rad Laboratories, Hercules, CA, USA). Images were captured using a Tanon5200 Imaging System (Biotanon, Shanghai, China). The band intensity was assessed with Image J software and normalized to that of GAPDH.

### 2.18 Statistical analysis

Statistical comparisons between two groups were performed using GraphPad Prism 8.00 software (GraphPad, CA, United States) by the unpaired *t*-test after confirming the normal distribution of the data by Anderson-Darling and Kolmogorov-Smirnov test. Differences for experiments with multiple groups were determined by one-way analysis of variance (ANOVA) followed by Tukey's multiple comparison test. Data were reported as means ± SEM. Results of statistically significant differences were denoted by asterisk. (P < 0.05 denoted by *, P < 0.01 denoted by **, P < 0.001 denoted by ***, and P < 0.0001 denoted by ****).

## 3 Results

### 3.1 Melatonin supplementation prevented the maternal age-associated decline in the number of COC TZPs

**Fig. 1:**
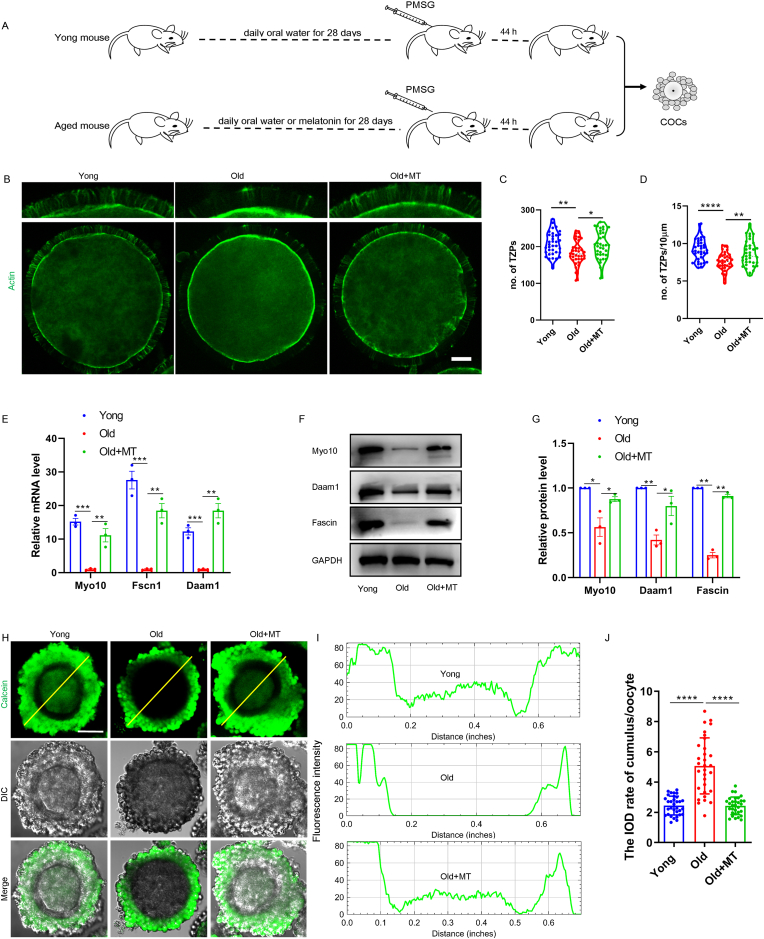
**Melatonin administration promotes generation of new TZPs.** (A) A timeline diagram of melatonin administration to mice and hormone injection for superovulation of oocytes. (B) Actin fluorescent staining represents the localization of phalloidin-stained TZPs in oocytes. Upper panels show enlarged portion of corresponding lower panels. Scale bar = 10 μm. (C) and (D) The number and density of TZPs were determined from confocal images. n = 35 (young), 35 (old), 35 (old-melatonin) COCs examined over three independent experiments. (E) The indicated mRNAs for encoding key structural components of TZPs were quantified in the COCs relative to *Gapdh*. Results were normalized to aged COCs. (F) Proteins for generating key structural components of TZPs were quantified in the COCs by immunoblotting. A representative immunoblot is shown. (G) Proteins were normalized to GAPDH. (H) Representative images show the calcein dye transmition from cumulus cells to oocytes. Scale bar = 50 μm. (H) The fluorescence intensity distributions of calcein along the yellow line to reflect permeability of gap junction. (J) The integrated optical density (IOD) rate of cumulus/oocyte in mouse COCs to reflect permeability of gap junction. n = 32 (young), 31 (old), 31 (old-melatonin) COCs examined over three independent experiments. (For interpretation of the references to colour in this figure legend, the reader is referred to the Web version of this article.)

To examine the contact and communication between oocytes and surrounding cumulus cells, COCs were collected from young and aged mice, and filamentous (F-) actin was stained using phalloidin to label TZPs. Consistent with previously published studies [6], we observed that the number of TZPs was significantly reduced in the COCs derived from maternal aged oocytes. We first investigated whether melatonin supplementation would ameliorate aging-mediated reduction in the number of TZPs in COCs. For this purpose, 30 mg/kg of melatonin was administered every day to aged female mice for 28 consecutive days (Fig. 1A). As expected, administration of melatonin in aged mice remarkably increased the number and density of TZPs in COCs (Fig. 1B, C, D), suggesting that melatonin can prevent aging-mediated reduction in the number of TZPs. We subsequently examined the expression of genes associated with the key structural components of TZPs in COCs. We observed a significant increase in the mRNA levels of the *Myo10, Fscn1,* and *Daam1* in melatonin-administered aged mice (Fig. 1E). Consistent with the increased levels of mRNA, the protein levels of these genes were also significantly improved by melatonin administration (Fig. 1F). We next tested whether the reduction in the number of TZPs was associated with impaired communication between oocytes and surrounding cumulus cells. COCs were preloaded with calcein-acetoxymethyl (C-AM), a cell permeability ester derivative of a fluorescence indicator, which can permeate inside the cumulus cells and produce a gap-junction-permeable calcein fluorescent molecule (Fig. 1H). The calcein fluorescence intensities in the oocytes and surrounding cumulus cells were assessed. In COCs derived from aged mice, approximately no fluorescence was observed in the oocyte, indicating impairment in gap junction communication. In contrast, extensive transfer of fluorescence to the oocytes of mice administered with melatonin was observed, indicating that functional gap junction communication was retained (Fig. 1 I and J). These results indicate that melatonin can partially restore the contact and communication between the oocyte and cumulus cells in aged mice.

### 3.2 Melatonin supplementation decreased the rates of aging-related death of cumulus cells

**Fig. 2:**
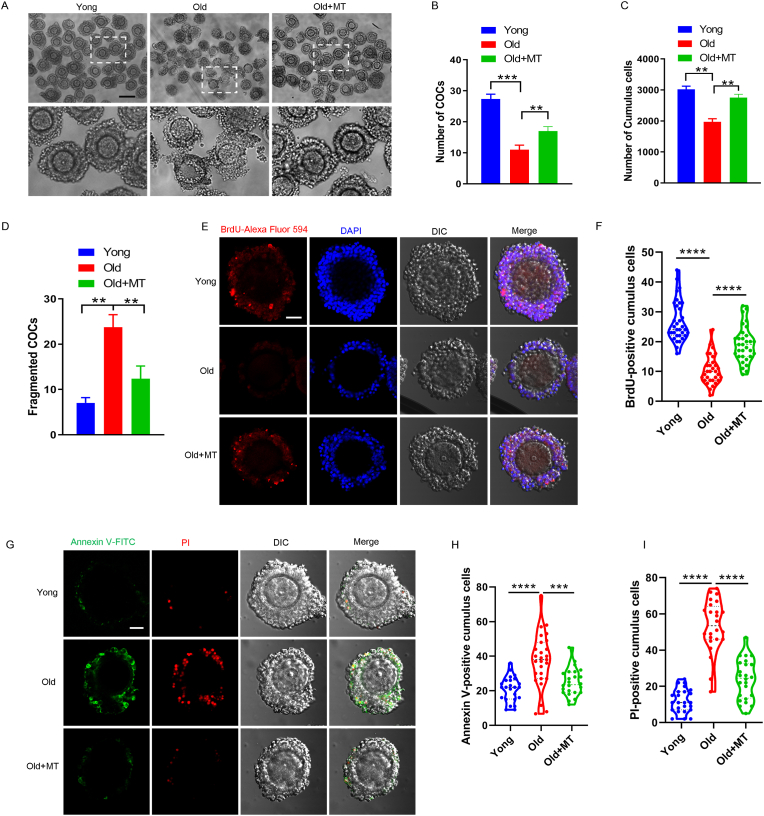
**Melatonin supplementation promotes cumulus cell number via regulation of apoptosis.** (A) Representative images show the morphological structure of COCs. Scale bar = 100 μm. (B) Number of COCs obtained from young, old, and old-Melatonin mice. (C) Number of cumulus cells per COCs. (D) Proportion of fragmented COCs from young, old, and old-Melatonin mice. (E) Representative images shown BrdU staining of COCs from young, old, and old-melatonin mice. Scale bar = 50 μm. (F) The number of BrdU-positive cumulus cells in an equatorial confocal optical section was counted. n = 33 (young), 31 (old), 31 (old-melatonin) COCs examined over three independent experiments. (G) Representative images shown Annexin V and Propidium Iodide (PI) staining of COCs from young, old, and old-melatonin mice. Scale bar = 50 μm. (H) The number of Annexin V-positive cumulus cells in an equatorial confocal optical section was counted. n = 24 (young), 27 (old), 24 (old-melatonin) COCs examined over three independent experiments. (I) The number of PI-positive cumulus cells in an equatorial confocal optical section was counted. n = 24 (young), 27 (old), 24 (old-melatonin) COCs examined over three independent experiments.

As TZPs are specialized filopodia that project from the surrounding cumulus granulosa cells reaching to the oocyte, we determined if age could compromise the physiological functions of cumulus cells. We evaluated the morphology of COCs and the number of cumulus cells surrounding each oocyte. The normal morphology of COCs and the number of cumulus cells per COC declined with aging. However, significant improvements were observed in these parameters after melatonin administration (Fig. 2A, B, C, D). To further investigate whether cumulus cell proliferation was promoted in mice administered with melatonin, the number of BrdU-positive cumulus cells was assessed (Fig. 2E). BrdU incorporation was more significantly impaired in aged COCs than that observed in the younger groups; however, it recovered following melatonin administration (Fig. 2F). To determine if age could compromise the physiological function of cumulus cells by inducing apoptosis, we performed annexin V/propidium iodide (PI) staining of COCs in young mice and old mice, including those who were administered melatonin and those who were not administered melatonin (Fig. 2G). The numbers of Annexin V and PI-positive cumulus cells were significantly increased in aged mice (Fig. 2H, I). As expected, melatonin administration decreased the number of Annexin V and PI-positive cumulus cells (Fig. 2H, I). Melatonin administration restored age-compromised physiological functions of cumulus cells by promoting cell proliferation, increasing the number of cumulus cells surrounding each oocyte, and decreasing the rates of cell death.

### 3.3 Melatonin mediated gap junctional coupling was required to attenuate ROS levels for suppressing DNA damage, apoptosis and ER stress in aged oocytes

**Fig. 3:**
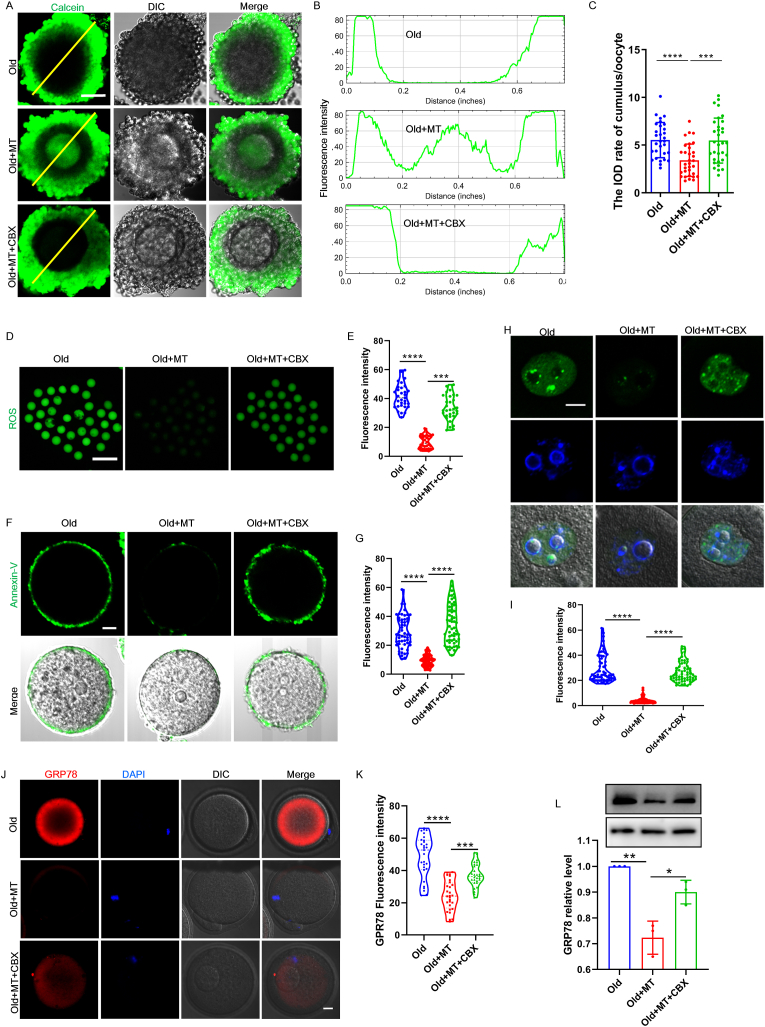
**CBX administration suppressed the effect of melatonin on ROS accumulation, DNA damage, and apoptosis in aged oocytes.** (A) Representative images show the calcein dye transmition from cumulus cells to oocytes. Scale bar = 50 μm. (B) The fluorescence intensity distributions of calcein along the yellow line to reflect permeability of gap junction. CBX supplementation suppressed the effect of melatonin on gap junctional communication. (C) The integrated optical density (IOD) rate of cumulus/oocyte in mouse COCs to reflect permeability of gap junction. n = 30(Old), 32 (Old + MT), 32 (Old + MT + CBX) COCs examined over three independent experiments. (D) Representative images show DCFHDA staining in aged (n = 31), melatonin + aged (n = 32), and melatonin + CBX + aged oocytes (n = 31). Scale bar, 100 μm. (E) The ROS fluorescence intensity was measured in aged (n = 31), melatonin + aged (n = 32), and melatonin + CBX + aged oocytes (n = 31). (F) Representative images of Annexin-V staining show apoptotic status in aged, melatonin + aged, and melatonin + CBX + aged oocytes. Scale bar, 10 μm. (G) The fluorescence intensity of Annexin-V signals were measured in aged (n = 53), melatonin + aged (n = 59), and melatonin + CBX + aged oocytes (n = 57). (H) Representative images stained with the γ-H2AX antibody show DNA damage in aged, melatonin + aged, and melatonin + CBX + aged oocytes. Scale bar, 10 μm. (I) The fluorescence intensity of γ-H2AX signals were counted in aged (n = 63), melatonin + aged mice (n = 60), and melatonin + CBX + aged (n = 62) oocytes. (J) Representative images stained with the GRP78 antibody show ER stress in aged, melatonin + aged, and melatonin + CBX + aged oocytes. Scale bar, 10 μm. (K) The fluorescence intensity of GRP78 signals were counted in aged (n = 29), melatonin + aged mice (n = 28), and melatonin + CBX + aged (n = 28) oocytes. (L) Western blot of GRP78 protein in oocyte from aged, melatonin + aged, and melatonin + CBX + aged mouse. (For interpretation of the references to colour in this figure legend, the reader is referred to the Web version of this article.)

The results showed that melatonin administration can partially restore the contact and communication between the oocyte and cumulus cells in aged mice. However, the effect of melatonin on gap junction communications was significantly inhibited in the COCs by blocking gap junctional activity by CBX injection in vivo (Fig. 3 A, B and C). The bidirectional gap junction communications are required for the metabolic coupling between oocytes and the surrounding somatic cells. Therefore, we performed dichlorofluorescein (DCFH) staining to measure the ROS levels in each group of oocytes (Fig. 3D). Fluorescence imaging and intensity measurements showed that melatonin supplementation effectively reduced ROS accumulation in aged oocytes (Fig. 3E). This observation may be attributed to the fact that melatonin prevents age-associated germline-soma communication deficits. As expected, pharmacological inhibition gap junction communication by administration of CBX effectively blocked the beneficial effect of melatonin on the ROS neutralization in aged oocytes (Fig. 3E). These results support the interpretation that gap junction communications are required to maintain metabolic coupling between oocytes and the surrounding cells.

As excessive ROS accumulation in aged oocytes typically results in apoptosis and oxidative damage in DNA [3], we next detected apoptotic cells by Annexin-V staining (Fig. 3F). The DNA damage was assessed using γ-H2A.X staining (Fig. 3H) in aged, melatonin + aged, and melatonin + CBX + aged oocytes. As expected, maternal aging led to apoptosis, and DNA damage signals were markedly decreased in the oocytes derived from aged mice that were administered melatonin compared to those observed in the melatonin + CBX group (Fig. 3 G and I). Oxidative burden is often linked to accumulation of misfolded proteins and ER dysfunction [36]. The ER function might be disturbed in the aged mouse oocytes. We then examined the ER stress level by analyzing the expression of ER stress marker protein GRP78. We found that melatonin effectively suppressed ER stress-associated GRP78 expression (Fig. 3 J, K and L). However, ER stress signals were markedly increased in the oocytes derived from melatonin + CBX treated mice (Fig. 3 J, K and L). Inhibiting gap junction communications thus blocks melatonin-rescued apoptosis, DNA damage and ER stress in aged mice.

### 3.4 Melatonin mediated gap junctional coupling was required for restoring age-related mitochondrial dysfunction and meiotic defects

**Fig. 4:**
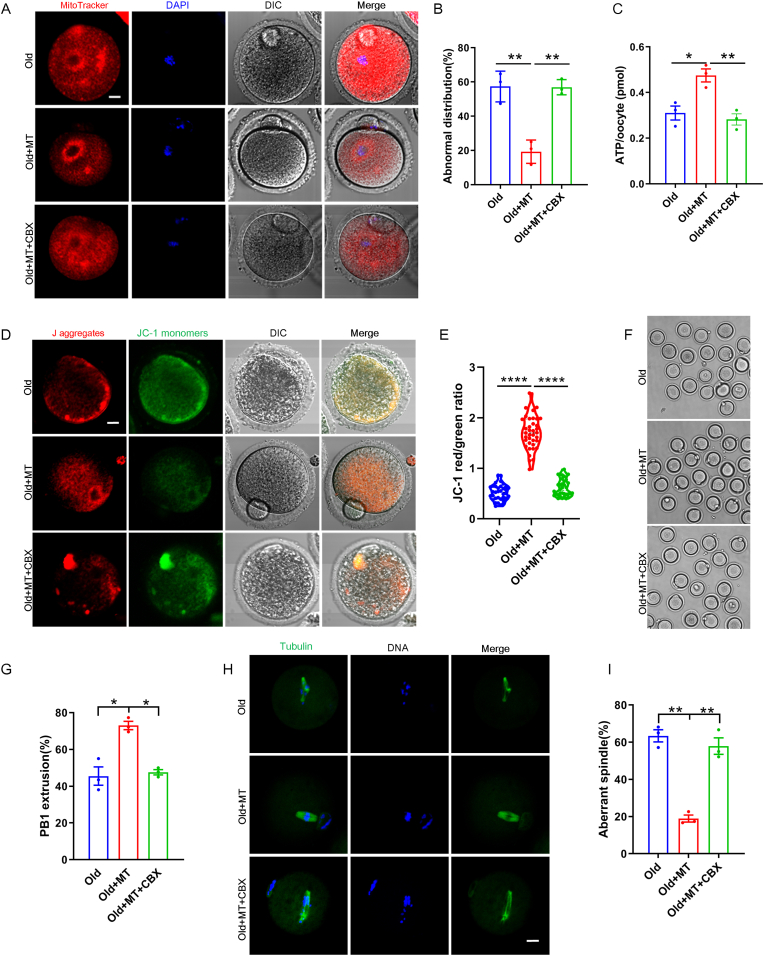
**CBX administration suppressed the effect of melatonin on mitochondrial distribution and function in aged oocytes.** (A) Representative images stained with MitoTracker Red show mitochondrial distribution in aged, melatonin + aged, and melatonin + CBX + aged oocytes. Scale bar, 10 μm. (B) The abnormal rate of mitochondrial distribution was recorded in aged, melatonin + aged, and melatonin + CBX + aged oocytes. (C) ATP levels were measured in aged, melatonin + aged, and melatonin + CBX + aged oocytes. (D) Representative images stained with JC-1 show mitochondrial membrane potential (ΔΨm) in aged, melatonin + aged, and melatonin + CBX + aged oocytes. Scale bar, 10 μm. (E) The ratio of red to green fluorescence intensity was calculated aged (n = 35), melatonin + aged (n = 37), and melatonin + CBX + aged oocytes (n = 34). (F) Representative images show morphology in aged, melatonin + aged, and melatonin + CBX + aged oocytes. Scale bar, 100 μm. (G) Proportion of PB1 in aged, melatonin + aged, and melatonin + CBX + aged oocytes. (H) Representative images show spindle morphology stained with α-Tubulin in aged, melatonin + aged, and melatonin + CBX + aged oocytes. Scale bar, 10 μm. (I) Proportion of aberrant spindle in aged, melatonin + aged, and melatonin + CBX + aged oocytes. (For interpretation of the references to colour in this figure legend, the reader is referred to the Web version of this article.)

To verify the effect of melatonin administration on mitochondrial function and oocyte maturation in aged oocytes, we examined mitochondrial distribution, ATP levels, mitochondrial membrane potential (ΔΨm), and aberrant spindle/chromosome structure. We observed aggregated distribution in the aged oocytes. However, abnormal distributions of mitochondria were significantly decreased in the melatonin-treated group, in which a homogeneous distribution of mitochondria was observed in the cytoplasm with accumulation in the periphery of chromosomes (Fig. 4A). Quantitatively, 57.36% ± 6.495% of aged oocytes demonstrated mislocalized mitochondria, and melatonin supplementation reduced this number to 19.27% ± 4.689% (Fig. 4B). In melatonin + CBX treated mice, however, mitochondria exhibited an aggregated distribution pattern in the cytoplasm with a significant absence around chromosomes (Fig. 4A and B). The important function of mitochondria, i.e., production of ATP, might be compromised with the abnormal distribution of mitochondria in aged oocytes. We measured ATP levels in oocytes derived from aged, melatonin + aged, and melatonin + CBX + aged mice. Our results showed that melatonin supplementation recovered ATP levels in aged oocytes, but ATP levels was prominently lowered in the oocytes derived from melatonin + CBX treated mice compared with those derived from aged mice administered only melatonin (Fig. 4C). Moreover, since the mitochondrial membrane potential is the driving force of ATP synthesis, we then assessed the mitochondrial membrane potential of oocytes via staining with JC-1 (Fig. 4D). As shown in Fig. 4E, mitochondrial membrane potential was significantly increased in melatonin-treated mice oocytes as compared with the control oocytes. However, it declined in oocytes derived from melatonin + CBX treated mice. The percentage of oocytes with first polar body (PB1) extrusion (Fig. 4F and G) and normal spindle (Fig. 4H and I) were also significantly increased in melatonin-treated aged mice. Melatonin + CBX treatment significantly reduced the ratio of normal MII oocytes and normal spindle compared to that observed with only melatonin treatment. These results suggested that disrupting the bidirectional communications between oocyte and somatic cells compromised the beneficial effects of melatonin on mitochondrial function and meiotic maturation.

### 3.5 Melatonin supplementation in vitro prevented the retraction of TZPs in COCs derived from aged mice

**Fig. 5:**
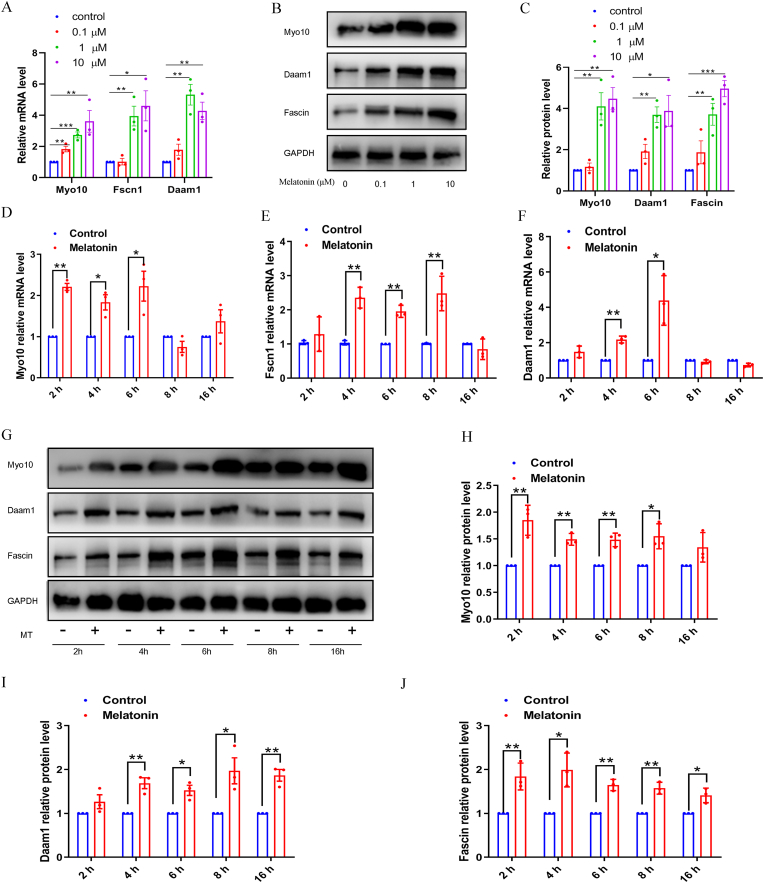
**Melatonin supplementation in vitro inhibited retraction of TZP in COCs from aged mice.** (A) The indicated mRNAs for encoding key structural components of TZPs were quantified in the COCs relative to *Gapdh*. Results were normalized to no melatonin supplementation aged COCs. (B) Proteins for generating key structural components of TZPs were quantified in the COCs by immunoblotting. A representative immunoblot is shown. (C) Proteins were normalized to GAPDH. (D) (E) (F) Following melatonin supplementation for 16 h, the mRNAs for encoding key structural components of TZPs were quantified in the COCs relative to *Gapdh*. (G) The proteins for generating key structural components of TZPs were quantified in the COCs by immunoblotting at the indicated times after addition of melatonin. A representative immunoblot is shown. (H) (I) (J) Proteins were normalized to GAPDH.

TZPs and gap junction communication is decreased in COCs in aged mice, and *Myo10, Fscn1, and Daam1* are associated with the structural components of TZPs. To determine whether supplementation of melatonin in vitro can prevent the retraction of the structural components of TZPs in aged maternal oocytes, we incubated COCs with melatonin in a medium for 6 h and then assessed the expression of *Myo10, Fscn1,* and *Daam1* under in vitro conditions. Melatonin receptors expression was detected in the granulosa cell, cumulus cell and oocyte (Figs. S1A and B). Treatment of COCs with 1 μM melatonin resulted in significant increases in the mRNA levels of *Myo10, Fscn1,* and *Daam1* (Fig. 5A). We also analyzed the expression of the three proteins. Consistent with the increased levels of mRNA, the protein levels were also significantly improved by melatonin supplementation (Fig. 5B and C). The dose-responsive studies identified 1 μM to be the effective concentration of melatonin. We then used the dose of 1 μM of melatonin for our subsequent in vitro examination. To investigate the mechanism of melatonin that drives the expression of structural components of TZPs in vitro, we incubated COCs in the presence of 1 μM melatonin for various intervals. After 4 h of incubation, the COCs showed significantly increased mRNA levels (Fig. 5D, E, F). We also observed increased protein levels in COCs incubated with melatonin, which also coincided with the kinetics of mRNA levels (Fig. 5G, H, I, and J). Furthermore, the expression of *Myo10*, *Fscn1*, and *Daam1* in the melatonin plus luzindole (a preferential melatonin receptor antagonist) treated COCs were significantly decreased as compared with those in the melatonin group (Fig. S2). These results indicated that exposure to exogenous melatonin stimulated the expression of the structural component proteins of TZPs in aged COCs during in vitro incubation, which may contribute to prevent the retraction of TZPs.

### 3.6 Oocyte-secreted factors participate in melatonin prevented TZP retraction

**Fig. 6:**
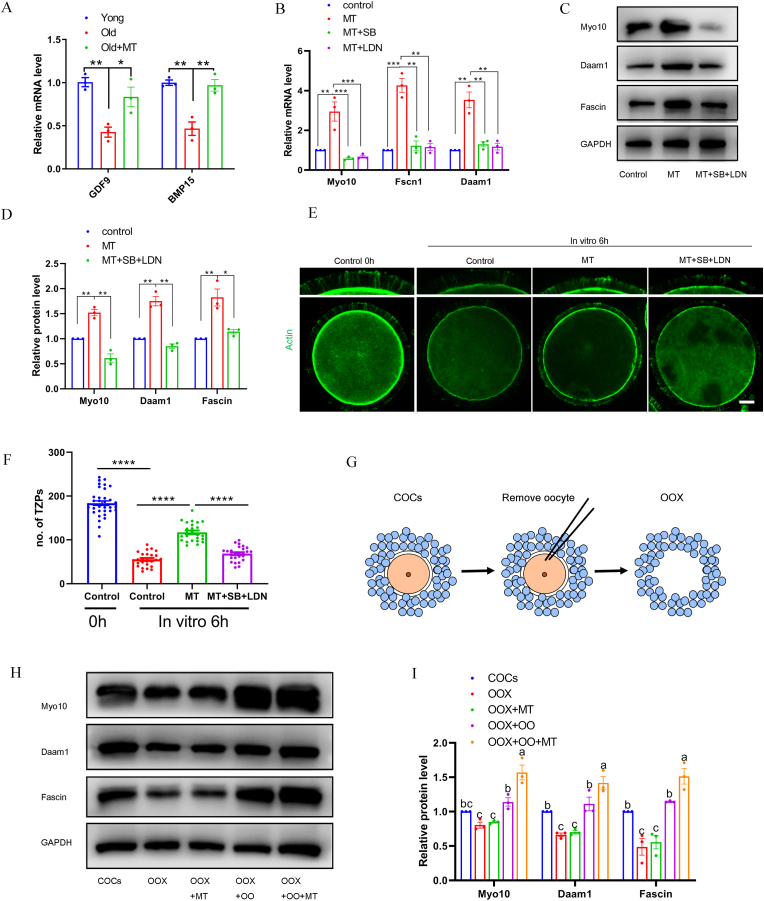
**Oocyte-secreted factors participate in melatonin prevented TZP retraction.** (A) The indicated mRNAs were quantified in the oocyte obtained from young, old, and old-Melatonin mice. Results were normalized to oocyte obtained from young mice. (B) Aged COCs were incubated in the indicated conditions. MT, melatonin; SB, SB431542; LDN, LDN193189. The indicated mRNAs were quantified in the COCs relative to *Gapdh*. Results were normalized to culture in the control. (C) Aged COCs were incubated in the indicated conditions. The proteins for generating key structural component of TZPs were quantified in the COCs by immunoblotting. A representative immunoblot is shown. (D) Proteins were normalized to GAPDH. (E) Actin fluorescent staining represents the localization of phalloidin-stained TZPs in oocytes. Upper panels show enlarged portion of corresponding lower panels. Scale bar = 10 μm. (F) The number of TZPs were determined from confocal images. n = 25 (control), 25 (MT), 26 (MT + SB + LDN) COCs examined over three independent experiments. (G) Oocytectomy procedure. Oocytes were removed from COCs, and the cumulus cell shells (OOX) were harvested. (H) The proteins for generating key structural components of TZPs were quantified in the indicated groups. A representative immunoblot is shown. (I) Proteins were normalized to GAPDH.

Considering the influence of the oocyte-secreted factors on the structure of TZP, the influence of melatonin on the expression of oocyte-secreted factors GDF9 and BMP15 was examined. We found that GDF9 and BMP15 mRNA levels in oocyte from aged mice were remarkably lower than those from young mice (Fig. 6A), indicating that the expression of oocyte-secreted factors decreases with age. Administration of melatonin in aged mice remarkably increased the mRNA levels in oocyte (Fig. 6A). After incubation of COCs for 6 h in the presence of melatonin and SB431542 and LDN193189 (inhibitor of the GDF9 and BMP15 pathway) in vitro, we analyzed the mRNA expression of *Myo10, Fscn1,* and *Daam1*. Melatonin increased the mRNA expression of *Myo10, Fscn1,* and *Daam1*, which was suppressed by SB431542 and LDN193189 (Fig. 6B). Incubation of COCs for 6 h in the presence of melatonin also increased the amount of all three proteins. In contrast, the incubation of complexes in the presence of SB431542 and LDN193189 led to a reduction in the amounts of the three proteins (Fig. 6C and D). We then proceeded with actin fluorescence staining to examine the localization of TZPs in maternally aged oocytes (Fig. 6E). Quantification of the number of TZPs revealed that the COCs cultured in melatonin medium possessed significantly more intact TZPs than the aged controls (Fig. 6F). Although numbers of TZPs in both groups subsequently declined, significantly more TZPs remained intact in the melatonin-cultured aged COCs than that in the control group at 6 h (Fig. 6F). In contrast, the incubation of COCs in the presence of SB431542 and LDN193189 led to a reduction in the number of TZPs (Fig. 6F). These observations suggested that inhibiting the SMAD activity blocked the effect of melatonin on TZP retraction.

We further investigated whether oocytes are required for the melatonin-mediated expression of *Myo10, Fscn1,* and *Daam1*. Oocytes were micro-surgically removed from COCs (oocytectomy, OOX) (Fig. 6G), and oocytectomized cumulus cell complexes were cultured with melatonin and/or oocytes (OO) for 6 h. In OOX cumulus cells or OOX cumulus cells maintained with melatonin alone, expression levels of the proteins associated with the TZP were significantly lower than those in the COCs. OOX cumulus cells maintained with oocyte alone demonstrated levels similar to those observed in COCs. Supplying both melatonin and oocyte to OOX cumulus cells significantly enhanced the expression of all three proteins (Fig. 6H, I). These results strongly suggest that the oocyte-derived GDF9 and BMP15 are required for the melatonin-stimulated expression of TZP structural proteins and prevention of TZPs retraction.

### 3.7 Melatonin supplementation in vitro combats oxidative stress by maintaining G6PDH activities and NADPH and GSH levels in aged COCs

**Fig. 7:**
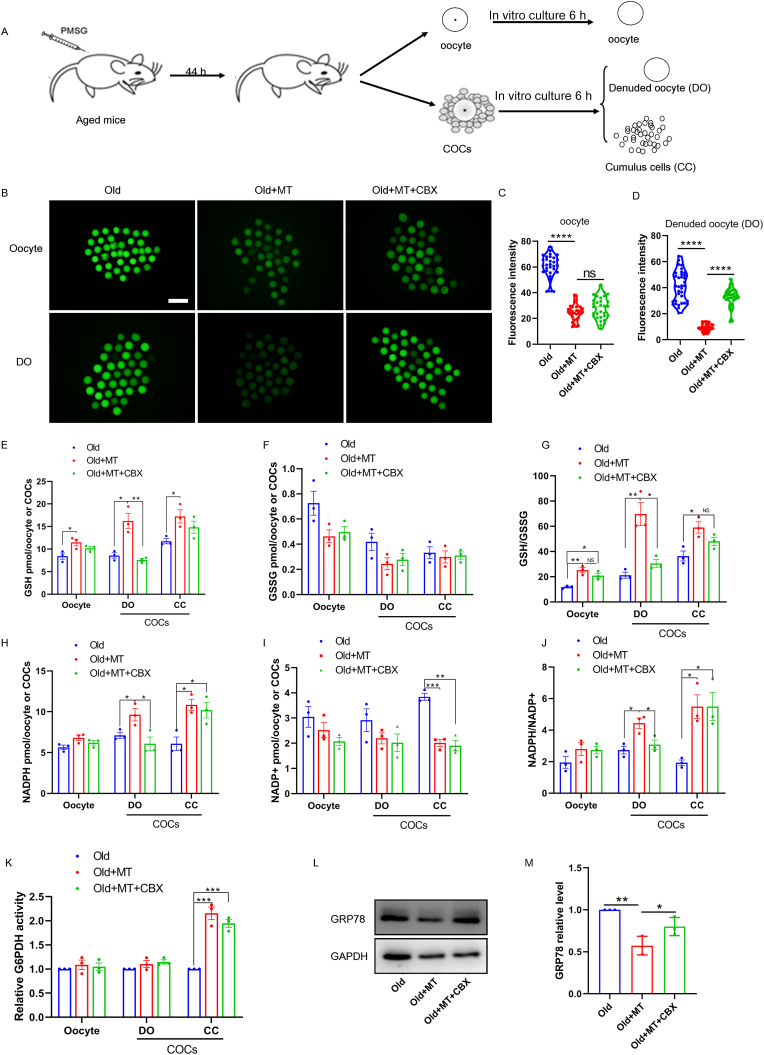
**In vitro melatonin treatment combat oxidative stress by maintain G6PDH activities, NADPH and GSH levels in aged COCs.** (A) A timeline diagram of hormone injection to mice for separation of oocytes and COCs and melatonin treatment in vitro. (B) Representative images show DCFHDA staining in aged oocytes, COCs (n = 32), and COCs denuded oocyte (DO). Scale bar, 100 μm. (C) The ROS fluorescence intensity was measured in aged (n = 29), melatonin + aged (n = 32), and melatonin + CBX + aged oocytes (n = 30). (D) The ROS fluorescence intensity was measured in aged (n = 32), melatonin + aged (n = 35), and melatonin + CBX + aged (n = 33) COCs denuded oocyte. Effect of melatonin on oocyte and COCs GSH levels, (F) GSSG levels and (G) GSH/GSSG ratio after 6 h of incubation. Effects of melatonin on oocyte and COCs (H) NADPH levels, (I) NADP + levels and (J) NADPH/NADP + ratios after 6 h of incubation. (K) Effects of melatonin on oocyte and COCs G6PDH activities after 6 h of incubation. (L) Western blot of GRP78 protein in oocyte from aged, melatonin + aged, and melatonin + CBX + aged mouse. (M) Proteins were normalized to GAPDH.

Gap junction communication between germ and somatic cells is crucial for the growth and maturation of competent oocytes as it provides necessary nutrients and signals to the oocytes. To demonstrate whether supplementation of melatonin can improve the quality of maternally aged COCs in vitro by eliminating excessive ROS levels, we treated the oocytes with melatonin in an in vitro maturation medium for 6 h and then evaluated the level of ROS in aged oocytes (Fig. 7A). As expected, we found that treatment of COCs with melatonin alone instead of melatonin with CBX significantly decreased the ROS signals, as observed in immunostaining assay and fluorescence intensity data (Fig. 7B, C, D). However, CBX did not affect the ROS signals of the oocyte when the cumulus cells were removed. The association of the reduction in gap junction communication in aged COCs with increased oocyte ROS production remains known.

One of the key functions of gap junction communication between oocytes and cumulus cells is to transmit metabolites, including the ROS scavengers, from the surrounding fluid following secretion by the cumulus cells to the oocytes [16]. Glucose-6-phosphate dehydrogenase (G6PDH) plays an important role in catalyzing the first reaction of the pentose phosphate pathway (PPP) for generating NADPH, an important cofactor in many cellular reactions, particularly for maintaining the cellular pool of GSH. GSH serves as one of the antioxidants in the oocytes to combat ROS oxidative stress. We then investigated if ROS scavenger metabolism was affected by aging. Our results show that both the GSH levels and GSH/GSSG ratio of the oocytes in the melatonin-supplemented medium were significantly higher than those observed in the oocytes in the control medium during 6 h of incubation (Fig. 7E, F, G). The gap junction inhibitor CBX decreases the GSH levels and GSH/GSSG ratio of oocytes derived from COCs after 6 h of incubation (Fig. 7E, F, G). Moreover, when we analyzed the NADPH level and NADPH/NADP + ratio of the oocytes after 6 h of incubation, both were significantly lower in the oocytes incubated in the control medium than that of the oocytes incubated in the melatonin-supplemented medium. The highest NADPH levels and NADPH/NADP + ratios were observed in oocytes derived from COCs and cumulus cells that were cultured in the melatonin-supplemented medium (Fig. 7H, I, J). To elucidate whether gap junction communication plays a role in maintaining NADPH levels in the oocyte, we incubated COCs with gap junction inhibitor CBX and melatonin. The results show that CBX decreases NADPH levels and NADPH/NADP + ratio of oocytes derived from COCs but not in cumulus cells after 6 h of incubation (Fig. 7H, I, J). Moreover, when we analyzed the G6PDH activity in the cumulus cells after incubation of COCs incubated in melatonin-supplemented medium, the activity was significantly higher than that in the COCs incubated in the melatonin-free medium (Fig. 7K). However, the oocyte G6PDH activity remained altered in all groups (Fig. 7K). The in vitro melatonin treatment led to the highest G6PDH activity in cumulus cells but not in the oocytes. These data suggested that both GSH and NADPH levels in COCs (primarily in cumulus cells) were improved by melatonin supplementation. Melatonin treatment reduced the relative GRP78 expression compared with controls, while the relative GRP78 expression was significantly increased after incubated COCs with gap junction inhibitor CBX compared with melatonin treatment (Fig. 7 L, M, Supplementary Fig. S3 A, B). These metabolites can be transmitted from cumulus cells to the oocytes, which led to the increase in NADPH/NADP+ and GSH/GSSG ratios, thereby reducing the oxidative burden and ER stress.

## 4 Discussion

In this study, we showed that the germline-soma communication and the metabolic flux of antioxidants decline with age, which may exacerbate oocyte apoptosis and senescence. Notably, we demonstrated that melatonin plays an important role in preventing TZP retraction, restoring the cumulus-oocyte communication, promoting cellular antioxidant mechanisms, and improving mitochondrial dynamics in aged mice, which is required for maintaining an optimal redox balance and oocyte quality. Moreover, boosting melatonin improves G6PDH activity and restores high NADPH/NADP+ and GSH/GSSG ratios in aged COCs, which reduces excessive ROS accumulation and prevents oxidative damage in oocytes.

During follicular development and oocyte growth, TZPs originate from granulosa cells and penetrate the zona pellucida. Some of them terminate at the oolemma to maintain a direct connection between oocytes and granulosa cells [15,37,38]. In growing oocytes, generation of numerous TZPs and establishment of gap junctions between granulosa cells and oocytes at the tip of TZPs permit the direct transfer of small metabolites, RNA, and signaling factors between oocytes and adjacent cells, ensuring proper oocyte development [12,13,37,39]. As demonstrated recently [6], defective oocyte-cumulus cell communication exacerbates the age-associated decline in oocyte quality. A more efficient or prolonged communication would allow a more potent transfer of metabolites and signaling factors from cumulus cells to the oocytes, supporting oocyte maturation and development [7,8,10]. In the present study, we showed that preventing TZP retraction and enhancing granulosa cell-oocyte communication could reduce maternal age-associated defects in oocytes.

The observation that melatonin supplementation rescues the morphology of COCs and increases the number of cumulus cells surrounding each oocyte suggests that proliferation of cumulus cells is required for maintaining morphological integrity and gap-junction coupling during follicle development. We proposed that melatonin supplementation leads to the remodeling of the metabolic framework balance that supports oocyte development by promoting the proliferation of cumulus cells and maintaining the communication coupling. Indeed, a large number of studies have shown that melatonin can be used to ameliorate oxidative stress-associated meiotic defects by protecting mitochondrial function in maternally aged oocytes [30,40,41]. An improved mitochondria function, in turn, act on melatonin synthetic enzymes, regulating melatonin biosynthesis and free radical scavenger, thus completing a feedback regulatory loop [25]. Our observations are perfectly aligned with these studies demonstrating the requirement of the restoration of mitochondrial dysfunction for melatonin-mediated enhancement of oocyte quality and the developmental competence [42]. The loss of oocyte the developmental competence is not only considered to be associated with the germ cells but also the defective granulosa cells in aged individuals [[43], [44], [45], [46]]. Our observations integrate the effects observed on the oocytes, surrounding cumulus cells, and gap-junction coupling between the oocytes and cumulus cells. We provided evidence that melatonin administration enhanced the expression of GDF9 and BMP15 in oocytes, which, in turn, promoted the proliferation of cumulus granulosa cells and prevented age-related TZP retraction, enabling more efficient transfer of metabolites from the cumulus cells. In accordance with recent investigation, loss of melatonin in maternal aged mice follicular fluid compromised oocyte quality by produce an excessive accumulation of ROS, DNA damage and ER stress [27,36]. We demonstrate an intriguing possibility that age-associated insufficiency of melatonin concentration in the follicular fluid microenvironment and defective germline-soma communication leads to the decline in oocyte quality.

Glucose metabolism in cumulus cells is essential for energy production and supply of pyruvate to the oocytes for ATP production [47]. The gap junction coupling architecture provides a functional channel for metabolites and ensures that the oocyte developmental needs are met [16]. The loss of this coupling with the granulosa cells has severe consequences for ATP production, meiotic progression, and oocyte developmental competence [39,42,48]. Here, we found that melatonin supplementation promotes ATP production in aged mice. However, blocking of gap junction coupling decreases both oocyte ATP levels and PB1 extrusion rates. Hence, the study further corroborates the theory that cumulus cells provide metabolic support via gap junctional communication to maintain mitochondrial function and support oocyte meiotic progression and developmental competence. Consistent with our findings, Richani et al. recently reported that the ATP levels increase in oocytes cultured with cumulus cells, and pharmacological inhibition of their gap-junction communication leads to a reduction of ATP levels in the oocytes [49]. These results demonstrate that the metabolic cooperativity between cumulus cells and oocytes mediated via gap junction communication directly affects intra-oocyte ATP levels.

The age-dependent accumulation of ROS is thought to exacerbate oxidative damage in oocytes [50]. A recent observation indicates that the activation of the Sirt1/Sod2 (superoxide dismutases 2) pathway by melatonin eliminates the excessive ROS accumulated in aged maternal oocytes, thus improving oocyte quality [27]. Similarly, our results also show that melatonin could decrease ROS levels in the oocyte in the absence of cumulus cells. Since glutathione cycles between the reduced (GSH) and oxidized (GSSG) form, electrons from GSH are important for the elimination of ROS in mouse oocytes. Down-regulation of GSH/GSSG in oocytes from older females may lead to ROS accumulation and oxidative damage. The cellular pool of GSH is maintained by NADPH that is produced via PPP [51]. G6PDH plays an important role in catalyzing the first reaction of PPP for generating NADPH. In our study, the G6PDH activity was enhanced upon incubation of COCs with melatonin. Consequently, the NADPH/NADP+ and GSH/GSSG ratios were also increased in response to melatonin. Moreover, COCs cultured in vitro with melatonin exhibited higher levels of NADPH and GSH than oocytes cultured without their cumulus vestment. It is worth noting that COCs treated with gap junction inhibitor CBX showed decreased GSH/GSSG and NADPH/NADP + ratios in the oocytes but not in cumulus cells. Thus, it is plausible that gap junction inhibition reduces antioxidants in oocytes by restricting the metabolite transfer from cumulus cells. The G6PDH activity of COCs elevated by melatonin appeared to induce metabolic alterations in cumulus cells, which increased the levels of antioxidants NADPH and GSH, enhanced antioxidant defense systems and ROS scavenging capacity, and reduced oxidation-associated damage accumulation [3,52,53]. Our results corroborate the melatonin antioxidant capacity in oocytes and demonstrate that melatonin indirectly enhances the delivery of cumulus-derived antioxidant factors.

In conclusion, this study sheds light on the valuable efficiency of melatonin in sustaining gap junction communication between the oocyte and cumulus cells by increasing the levels of mRNAs and proteins corresponding to the genes associated with key structural components of TZPs. Such prolonged gap-junction communication enables the continued transfer of metabolites and leads to a decrease in intra-oocyte ROS levels, which might explain the improvement in oocyte quality. Also, a sufficient supply of intracellular antioxidants NADPH and GSH in aged COCs treated with melatonin due to the increased activities of G6PDH was observed. These findings contribute to our understanding of the mechanisms underlying age-associated decline in oocyte quality. A sufficient supply of intracellular antioxidants in oocytes may be implicated in promoting the activity of ROS scavenging systems. These results are important as they provide a new perspective on the mechanisms of melatonin that benefit reproductive physiology, which support the theoretical basis for the application of melatonin to enhance oocyte quality and improve female fertility during reproductive aging.

## Ethics approval

The experimental protocols and mice handling procedures were reviewed and approved by the Institutional Animal Care and Use Committee of the College of Veterinary Medicine, Northwest A&F University (No. 2018011212).

## Author contributions

Hui Zhang and Chan Li conceived the study, performed experiments and writing the manuscript. Dongxu Wen, Ruoyu Li, Sihai Lu, Rui Xu, Yaju Tang, Yidan Sun, Xiaoe Zhao and Menghao Pan performed experiments and collected data. Baohua Ma took the lead in writing the manuscript and was in charge of overall direction and planning. All authors provided helpful discussion and helped shape the research, analysis, and manuscript.

## Orcid

Hui Zhang 0000-0001-8893-0234.

Baohua Ma 0000-0002-8332-4275.

## Declaration of competing interest

All of the contributing authors declared no conflicts of interest.

## Data Availability

The data used to support the findings of this study are available from the corresponding author upon request.
