# Supplementary material for: Melatonin improves the quality of maternally aged oocytes by maintaining intercellular communication and antioxidant metabolite supply
Source: Redox Biol. 2021 Dec 17;49:102215. doi: 10.1016/j.redox.2021.102215 (PMC8688718; doi:10.1016/j.redox.2021.102215)
Supplement: Multimedia component 1 [file mmc1.docx]

**Supplementary Information**

**
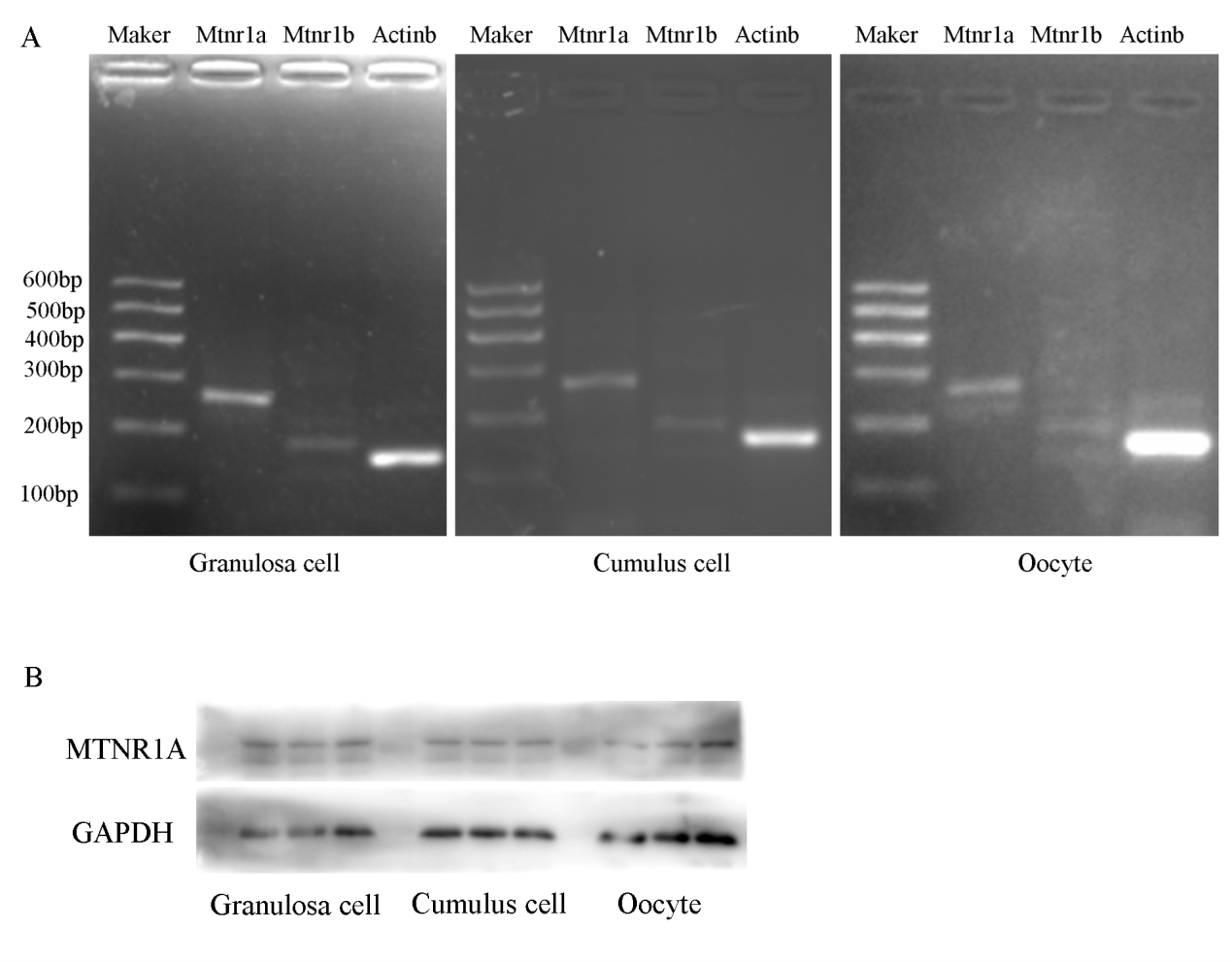
**

**Figure S1. The expression of melatonin receptors in granulosa cell, cumulus cell and oocyte from maternally aged mice.**

(A) Detection of melatonin receptors Mtnr1a and Mtnr1b transcription by RT-PCR in isolated granulosa cells, cumulus cells, and oocytes. (B) Western blot analysis showing detection of MTNR1A protein in maternally aged mouse granulosa cells, cumulus cells, and oocytes. GAPDH was used as a control to correct for loading.


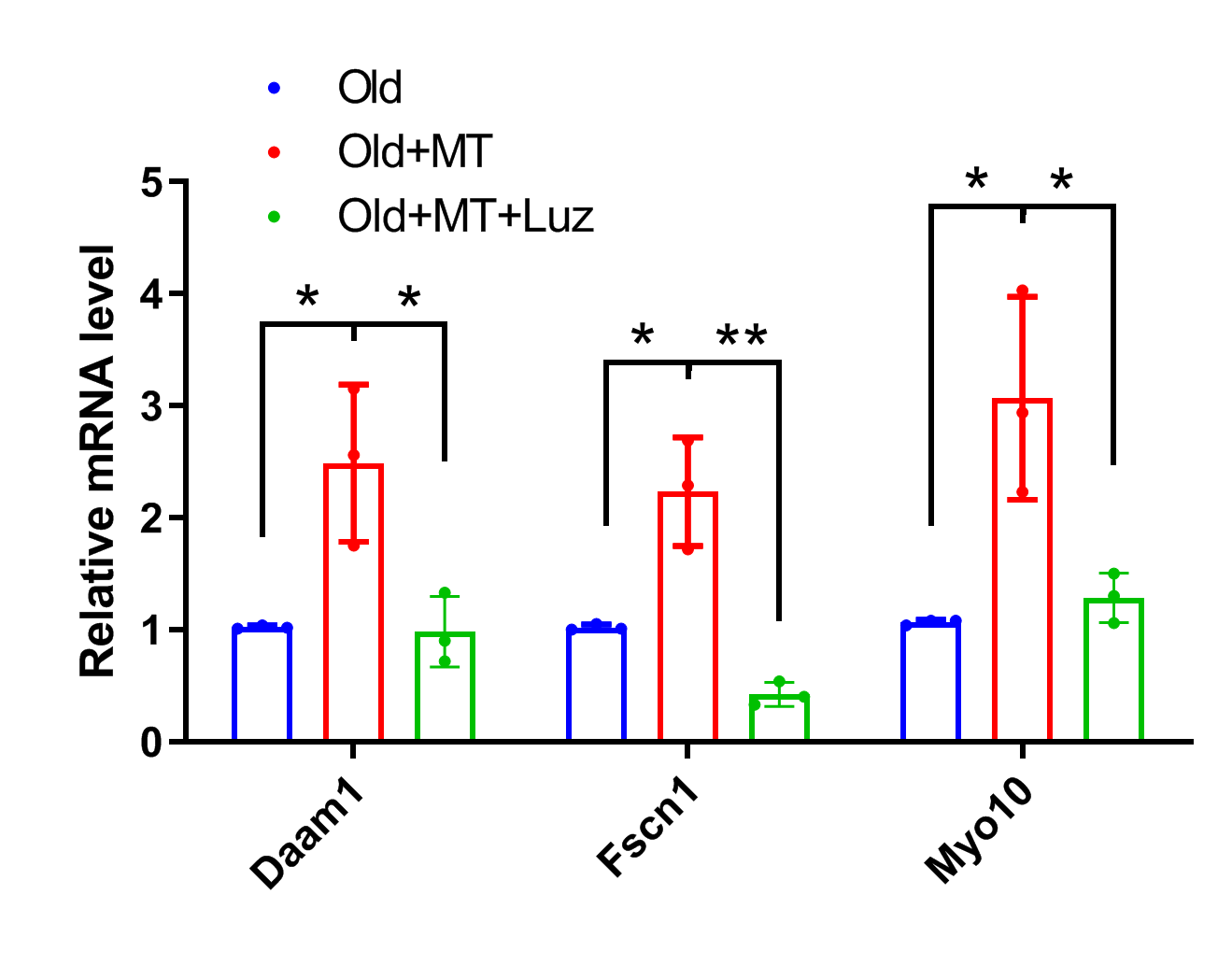


**Figure S2. Melatonin induced expression of mRNAs for encoding key structural components of TZPs in COCs from maternally aged mice.**

The indicated mRNAs for encoding key structural components of TZPs were quantified in the COCs relative to *Gapdh*. Quantifed data shown as mean± SEM of fold changes relative to levels in the Old group. **P* < 0.05, ***P* < 0.01. MT, Melatonin; Luz, luzindole, a preferential melatonin receptor antagonist.


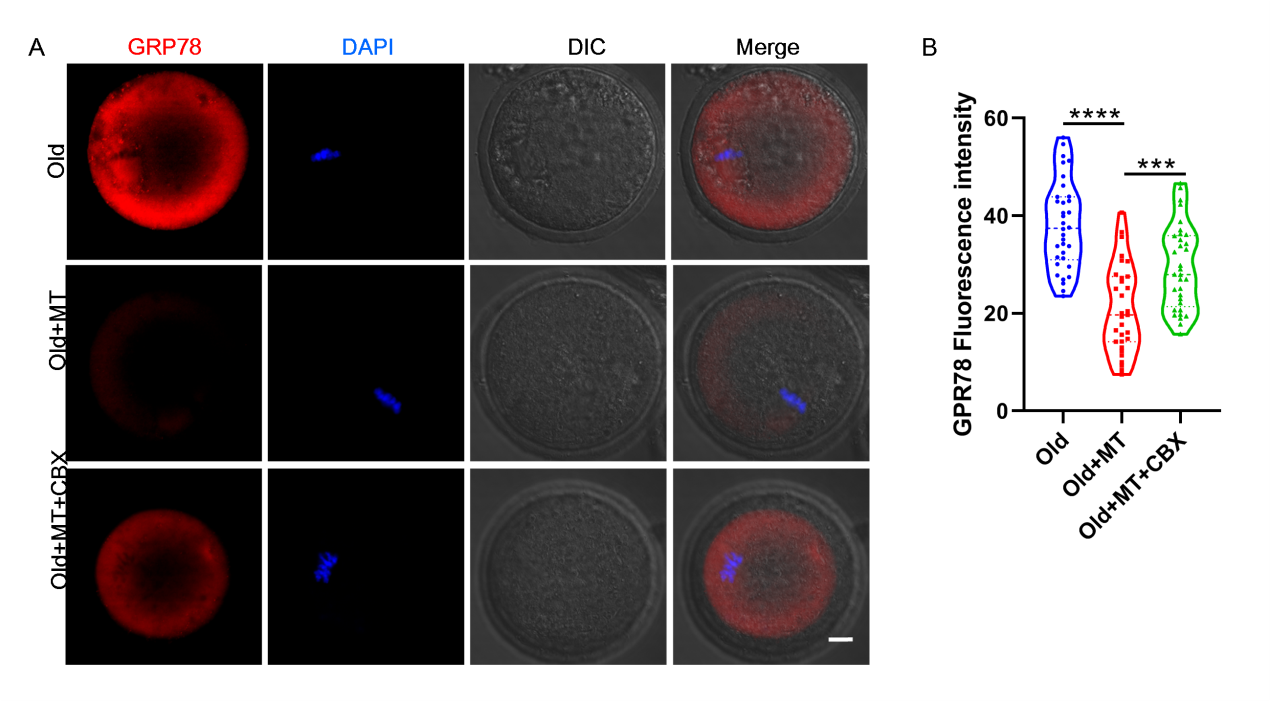


**Figure S3. Effects of melatonin treatment *in vitro* on the GRP78 levels.**

(A) Representative images stained with the GRP78 antibody show ER stress in aged, melatonin+ aged, and melatonin+ CBX+ aged oocytes. Scale bar, 10 μm. (B) Fluorescence intensity of GRP78 signals were measured in aged (n=34), melatonin+ aged (n=31), melatonin+ CBX treated (n=33) oocytes. ****P* < 0.001, *****P* < 0.0001..
